# Characterization of Insulin Antibodies by Surface Plasmon Resonance in Two Clinical Cases: Brittle Diabetes and Insulin Autoimmune Syndrome

**DOI:** 10.1371/journal.pone.0084099

**Published:** 2013-12-30

**Authors:** Aldana Trabucchi, Ruben F. Iacono, Luciano L. Guerra, Natalia I. Faccinetti, Andrea G. Krochik, María C. Arriazu, Edgardo Poskus, Silvina N. Valdez

**Affiliations:** 1 School of Pharmacy and Biochemistry, University of Buenos Aires (UBA) and Prof. Ricardo A. Margni Humoral Immunity Institute (IDEHU), National Research Council (CONICET)-UBA, Buenos Aires, Argentina; 2 Nutrition Service, J. P. Garrahan National Pediatrics Hospital, Buenos Aires, Argentina; 3 Pedriatrics Service, Private Community Hospital, Mar del Plata, Argentina; Kaohsiung Chang Gung Memorial Hospital, Taiwan

## Abstract

In this study, the characterization of insulin (auto)antibodies has been described, mainly in terms of concentration (q), affinity (K_a_) and Ig (sub)isotypes by Surface Plasmon Resonance (SPR) in two particular clinical cases of individuals with severe episodes of impaired glycemia. Subject 1 suffers from brittle diabetes associated with circulating insulin antibodies (IA) due to insulin treatment. Subject 2 has insulin autoantibodies (IAA) associated with hypoglycemia in spite of not being diabetic and not having ever received exogenous insulin therapy. After conventional screening for IA/IAA by radioligand binding assay (RBA), we further characterized IA/IAA in sera of both patients in terms of concentration (q), affinity (K_a_) and Ig (sub)isotypes by means of SPR technology. In both cases, q values were higher and Ka values were lower than those obtained in type 1 diabetic patients, suggesting that IA/IAA:insulin immunocomplexes could be responsible for the uncontrolled glycemia. Moreover, subject 1 had a predominat IgG_1_ response and subject 2 had an IgG_3_ response. In conclusion, SPR technology is useful for the complete characterization of IA/IAA which can be used in special cases where the simple positive/negative determination is not enough to achieve a detailed description of the disease fisiopathology.

## Introduction

Circulating Insulin antibodies (IA) are often detected in diabetic patients undergoing insulin treatment, however, these antibodies rarely interfere with the therapy and/or are associated with hypoglycemic or hyperglycemic episodes. However, a subset of insulin-treated patients with extremely high levels of IA are insulin resistant, with mean insulin binding capacities greater than 216 nM (30,000 microunits of insulin/ml serum) [1]. Ishizuka et al. [2] have described two cases of patients who produced low affinity and high insulin binding capacity of these antibodies while undergoing insulin treatment. These patients suffered from severe daytime hyperglycemia and early morning hypoglycemia which could be the result of massive volumes of insulin binding to the IA inducing hyperglycemia and later on, hypoglycemia due to the release of insulin from the immunocomplexes, [3]. Thus, brittle diabetes is the term used to describe uncontrolled type 1 diabetes which has been reported to occur in about 1 to 2% of patients who experience dramatic variation in blood glucose levels during the daytime. The glucose levels imbalance, in turn, leads to frequent episodes of keto-acidosis requiring that the patient be hospitalised [4], [5].

On the other hand, there are some cases where the episodes of hypoglycemia are a consequence of the presence of high levels of insulin autoantibodies (IAA) to endogenous insulin, despite never having received insulin injections. The Insulin Autoimmune Syndrome (IAS) is a well known example of the latter clinical status. This syndrome, first reported by Hirata et al. [6], has a strong association with HLA DR4 [7], [8] and with drug-induced autoimmunization caused by the administration of drugs containing sulphydryl groups (i.e. methimazol, thiamazol, glutathione or D-penicillamine) [9].

IA are routinely assessed by the Radioligand Binding Assay (RBA) first described by Kurtz and Nabarro [10], whereas IAA were first detected by an optimized RBA using mono (A14) [^125^I]-insulin as tracer [11]. When RBA signals exhibit high levels (e.g.: B%>20%) it is feasible to obtain the absolute parameters of the antibody:antigen interaction, by displacement Radioimmunoassay (RIA), using the conventional tracer or [^35^S]-Cysteine proinsulin [12]. Such parameters are the affinity constant (the median K_0_, for polyclonal antibodies, [13]) and the specific antibody concentration (q), usually expressed as binding capacity (BC). In this regard, Achenbach et al. [14] have carried out a workshop to assess whether four laboratories could reproducibly measure IAA affinity in coded sera from non-diabetic relatives of patients with type 1 diabetes, newly diagnosed patients, and healthy blood donors, and whether combining affinity with autoantibody titre could improve concordance and performance of IAA assays. This was evaluated by competitive binding using constant amounts of [^125^I]-insulin and increasing quantities of unlabeled human insulin.

The Surface Plasmon Resonance (SPR) technology is an alternative method to RIA to determine the primary interaction parameters. Moreover, these parameters can be measured in a real-time fashion. The biosensors based on SPR technology detect changes in the refraction index produced when an analyte (in this case antibodies) binds to its counterpart (in this case antigens) fixed on a sensor chip surface. This interaction can be expressed in terms of the kinetic association constant (k_1_) and kinetic dissociation constant (k_-1_), and also in terms of equilibrium affinity constant (K_a_), where K_a_ = k_1_/k_-1_.

In addition, by means of SPR it is possible to determine the Ig (sub)isotypes involved in the humoral immune response. The maturation of the immune response against insulin in preclinical type 1 diabetes has been assessed in sera samples from the Finnish Type 1 Diabetes Prediction and Prevention Study (DIPP), by observing the emergence of various isotypes of IAA in children with HLA-DQB1-conferred disease susceptibility. Results demonstrated that those children who progressed to type 1 diabetes had a dominant IgG_1,_ whereas IgG_3_ antibodies were more prevalent before the initiation of exogenous insulin therapy [15], [16].

The aim of the present study was to characterize IA/IAA in terms of concentration (q), affinity (K_a_) and Ig (sub)isotypes by SPR technology in two representative high titer IA/IAA sera from patients who presented brittle diabetes (Subject 1) or hypoglycemia episodes (Subject 2 with presumptive IAS) associated with such (auto)antibodies.

## Materials and Methods

### Case Report

Subject 1 is a 12-year-old Caucasian infant diagnosed with type 1 diabetes at 18 months of age. The patient had been administered exogenous insulin thenceforward, developing lipodystrophy in the places of injection. He presented brittle diabetes with a glycemia range of 55–400 mg/dl. At 4 years of age, the patient’s body mass index (BMI) was 16.5. The IA binding rate measured by RBA was B% = 48.2% (cut off value = 3.28%). Treatment was initiated with NPH human insulin but further changed to porcine insulin injected around the lypodystrophic sites, which improved lypoatrophy. Since glycemia was still uncontrolled, treatment varied over time from Glargina™ (17 U/day) to Levemir™ (21 U/day). Despite the changes in the therapeutic schedule, the episodes of fasting hypoglycemia recurred and hypoglycemic bouts started to occur after meals. After 10 years of evolution, the patient continued with regular metabolic control, lipodystrophy, BMI = 19 and HbA1c of 8.6%.

Subject 2 is at present a 27-year-old Caucasian woman who had never received insulin treatment nor thiol-related drugs. Since she was 10 years old she has presented hypoglycemic symptoms. At 11 years old her BMI was 15.3 and her current BMI is 21.9. IAA measured by RBA revealed high binding signals to insulin (B% = 62.1%, cut off value = 3.28%). All other type 1 diabetes humoral markers assayed (glutamic acid decarboxylase autoantibodies –GADA–, protein tyrosine phosphatase IA-2 autoantibodies –IA-2A– and zinc transporter 8 autoantibodies –ZnT8A–) were negative. Furthermore, DQB1 genotyping did not demonstrate the presence of alleles associated with type 1 diabetes susceptibility.

The initial treatment was 40 mg/day of methilprednisolone but gradually it was reduced to 2 mg/day since she had presented persistent episodes of hyperglycemia. When hypoglycemia reappeared, former doses of the corticoid were used. Eighteen months later, her treatment was switched to 50 mg of Acarbose previous meal time, together with the prescription of a diet without carbohydrates of rapid absorption. As the episodes of hypoglycemia and hyperglycemia continued to occur, she was subjected to plasmapheresis and treatment with the monoclonal antibody Rituximab™ without obtaining any improvement of her clinical condition. Since then, the time course of IAA levels has been systematically evaluated, showing no significant decrease despite the therapy used. Other potential endocrine abnormalities that could cause hypo- and hyperglycemia were ruled out.

### Control Group

Sera from 28 children and adolescents admitted to the Nutrition Service at the J. P. Garrahan Pediatric Hospital (Buenos Aires, Argentina), with a mean age of 8.31±4.20 at diagnosis were collected before or within 72 h of insulin treatment initiation. Type 1 diabetes was diagnosed according to WHO criteria [16]. The starting group included 71 children and adolescents attending the service from June 1994 to July 1996. As the hospital is a referral centre, patients came from all over Argentina and were mainly Caucasians. All these patients were tested in parallel for humoral markers of diabetes, IAA/proinsulin autoantibodies (PAA), GADA, IA-2A and ZnT8A. Most patients (71.8%) were GADA–positive. The second marker in frequency was ZnT8A (69.0%) followed by IA-2A (66.2%) and IAA/PAA (36.6%) (Table S1).

Blood samples were collected after overnight fasting and sera were stored at 20°C until assayed. The collection of serum samples from newly diagnosed type 1 diabetic patients, and the respective protocols were approved by the Ethical Committees of the J. P. Garrahan National Pediatric Hospital.

Written consent from all participants involved in this study as well as parental consent when being a minor was obtained.

### Radioligand Binding Assay (RBA)

(Auto)antibodies were first assessed by routinely screening using RBA [12] and fulfilling the criteria mentioned by Davidson and DeBra [1] based on BC of (auto)antibodies.

Briefly, cDNA coding for human proinsulin (PI) was transcribed and translated using a rabbit reticulocyte lysate system in the presence of [^35^S]cysteine (New England Nuclear, Boston, MA), according to the manufacturer’s instructions. After overnight refolding, favoured by a disulphide reduction-reoxidation procedure, [^35^S]-PI was isolated by reverse-phase HPLC. Sera (30 µl) were incubated for seven days at 4°C with 1,000 cpm of [^35^S]-PI in 90 µl of RBA buffer (50 mM sodium phosphate, 100 mM NaCl, pH 7, 0.1% Aprotinin and 0.1% bovine serum albumin). In order to isolate the immunocomplexes, 50 µl of a 50% suspension of Protein G-Sepharose 4B FF (Amersham Biosciences, Piscataway, NJ) in RBA buffer were subsequently added. Samples were centrifuged and supernatants were discarded. Pellets were washed four times with 200 µl of RBA buffer, suspended in 100 µl 1% SDS, and centrifuged (5 min at 6,000×*g*). Supernatants were carefully transferred to appropriate vials for scintillation counting, which was performed for 5 min each vial. Results were calculated as B% = 100×bound cpm/total cpm and expressed as *SDscore* = (B%–B_C_%)/SD_C_, where B_C_% was the mean B% of control sera and SD_C_ its standard deviation. Thirty normal control sera were included. B_C_% was normally distributed. An assay was considered positive if *SDscore*>3.

### Radioimmunoassay (RIA) and Binding Capacity (BC)

When B% values of IA/IAA were higher than 20%, RIAs together with conventional data processing were carried out in order to determine absolute values of IA/IAA in terms of concentration expressed as BC: insulin units per litre of serum (U ins/L serum) [17]. Values of BC>30 U ins/L serum were considered demonstrative of the involvement of hyperglycemic and hypoglycemic episodes [1].

### Surface Plasmon Resonance Analysis (SPR)

The assessment of antibody:antigen interaction parameters, q and K_a_, was performed as previously described [18]. All serum samples were centrifuged before injecting into the biosensor.

#### IA/IAA concentration

To evaluate q of IA/IAA, the experiments were carried out over a carboxymethylated-dextran CM5 sensor chip (GE Healthcare, Uppsala, Sweden) surface immobilized with standard proinsulin (Eli Lilly, Indianapolis, IN, USA) in an amount corresponding to 1600 Resonance Units (RU). The binding rates were measured after 120 s in running buffer (0.14 M NaCl, 2.7 mM KCl, 1.5 mM KPO_4_H_2_, 8.1 mM Na_2_PO_4_H, pH 7.4, 0.05% Tween 20). After each antigen-antibody interaction, a regeneration step was carried out using 10 mM glycine-HCl, pH 1.5. All sensorgrams were corrected by subtracting the signal from the reference flow cell.

A standard curve (RU *vs* antibody concentration) was prepared by using a rabbit anti-human-proinsulin polyclonal serum (300 nM determined by RIA). The concentrations used were: 75 nM, 37.50 nM, 18.75 nM, 9.38 nM, 6.69 nM, 2.34 nM, 1.17 nM and 0.59 nM. When measuring the binding rates, sera from both patients were diluted 1/10 and 1/30 in running buffer. For each sample the concentration of specific (auto)antibodies was derived from the standard curve. All experiments were carried out at 25°C with a flow rate of 10 µl/min.

#### IA/IAA affinity

For the determination of IA/IAA affinity, the sensor chip was prepared by using lower concentrations of immobilized antigen (300 RU). Assays were carried out at 20°C using a flow rate of 10 µl/min. A 1∶1 binding model was used.

**Figure 1:**
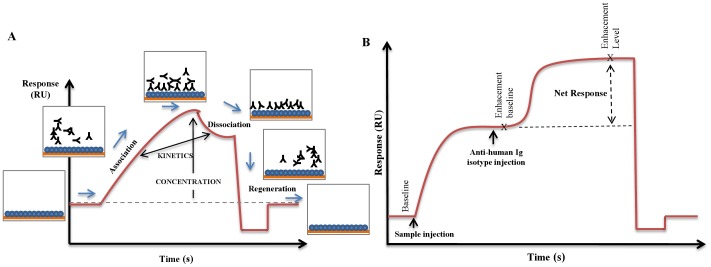
Representative sensorgrams obtained by SPR technology. *A*: Schematic representation of how antigen:antibody interaction parameters were calculated. *B*: Schematic representation of an Enhancement Assay to determine (sub)isotypes of specific Ig.

Each patient’s serum was used diluted 1/2, 1/4 and 1/8 in running buffer. In either case these starting samples were diluted 1/2 with carboxymethyl-dextran and NaCl to a final concentration of 1 mg/ml and 0.35 M, respectively, in order to eliminate nonspecific reactions. Each sample was injected over 300 s and the binding rate in running buffer was measured after 300 s. The association rate constant (k_1_), the dissociation rate constant (k_−1_), and the equilibrium constant (K_a_), were calculated from the sensorgrams analysis using the Biacore T100 Evaluation Software version 2.0 (Biacore® T100 Software Handbook) (Fig. 1*A*).

#### IA/IAA (Sub)isotyping

To perform the (sub)isotyping of Ig involved in the specific humoral immune response, each serum was analysed diluted 1/10 in running buffer, and injected during 300 s, over a sensor chip surface with proinsulin immobilized in an amount corresponding to 2000 RU. Anti-human isotype antibodies -anti-IgG, anti-IgM and anti-IgA- (DAKO Denmark S/A, Glostrup, Denmark) and anti-human IgG subisotype antibodies -anti-IgG_1_, anti-IgG_2_, anti-IgG_3_, anti-IgG_4_ (BD Biosciences Pharmingen, San Diego, CA, USA) were subsequently injected diluted 1/50 in running buffer over 120 s, previous the regeneration step. All sensorgrams were corrected by subtracting the signal from the reference flow cell. Net response in RU was informed as the difference between enhancement level and basal enhancement level (Fig. 1*B*).

## Results

In this work, we have analysed two particular clinical cases of patients with high IA/IAA titre together with severe uncontrolled glycemia. As described previously, subject 1 was a boy with brittle diabetes and lipodystrophy in insulin injection sites associated to the presence of IA. Subject 2 had recurrent hypoglycemic episodes related to the presence of IAA, but he had never had neither signs nor clinic history of type 1 diabetes.

(Auto)antibody titers were first determined by conventional RBA and expressed as B% and SD score. In addition, RIA with Scatchard analysis was performed (Table 1).

**Table 1:** Comparative IA/IAA data achieved with sera from subjects 1 and 2.

			Subject 1	Subject 2	Type 1 Diabetes
*A*	**RBA**	**Signal (B%)**	48.2	61.2	11.69±9.02^a^
		**Precision unit (SDs)**	26.72	46.75	12.48±1.748^a^
*B*	**RIA/Scatchard**	**BC (U/L)**	76	195	ND
*C*	**SPR**	**k_1_ (×10^5^ M^−1^s^−1^)**	36.8	5.70	ND
		**k_-1_ (s^−1^)**	0.36	0.17	ND
		**q (×10^−9 ^M)**	250.55	352.25	85.52±9.980^a^
		**K_a_ (×10^6^ M^−1^)**	10.30	3.37	105.40±31.70^a^

*A*: Conventional data: B% and SD score (SDs) obtained from RBA signals; *B*: Binding Capacity (BC) determined by RIA and Scatchard processing; *C:* SPR derived parameters: kinetic constants (k_1_ and k_-1_), concentration (q) and affinity (K_a_) calculated by the BIAevaluation software (BIAcore™).

^a^ Mean values calculated from 28 IAA positive type 1 diabetic patients.

ND: Not Determined.

### IA/IAA Concentration and Affinity

**Figure 2:**
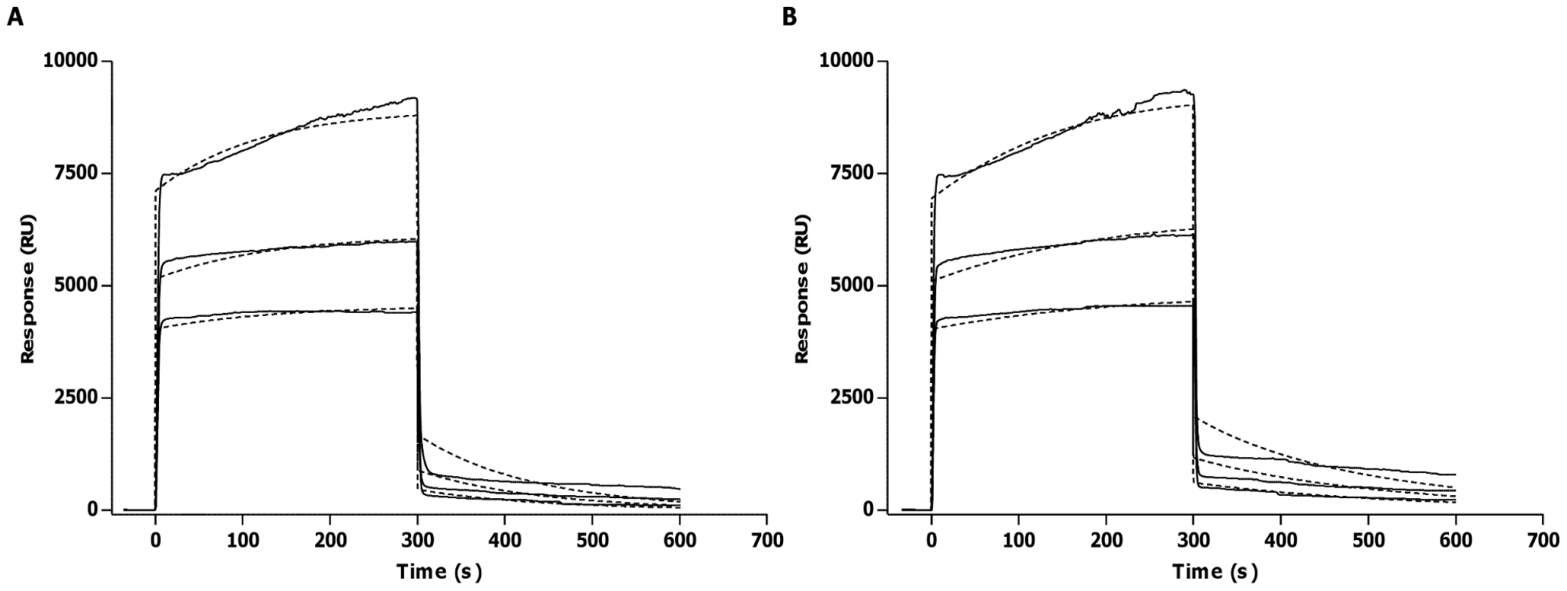
Fitting of experimental data (full line) to theoretical binding curves (dotted lines) in the affinity measurements of specific (auto)antibodies. *A*: IA affinity measurements of subject 1. *B*: IAA/PAA affinity measurements in subject 2.

By means of SPR technology it was possible to determine q and K_a_ of the specific (auto)antibodies. The q value of IA present in subject 1 serum was 250.55×10^−9 ^M, while q value of IAA in subject 2 serum was 352.25×10^−9 ^M. Both values were significantly high when compared to those of sera from type 1 diabetic patients. Regarding K_a_ values, these were 10.3×10^6 ^M^−1^ and 3.37×10^6 ^M^−1^ for subject 1 and 2, respectively, and were significantly lower than those found in type 1 diabetic patients. Figure 2 shows fits of the experimental curves to theoretical binding curves generated by BIA-evaluation software.

The results concerning concentration and affinity of both subjects studied compared to type 1 diabetic patients are summarized in Table 1.

### IA/IAA Isotyping and Subisotyping

**Figure 3:**
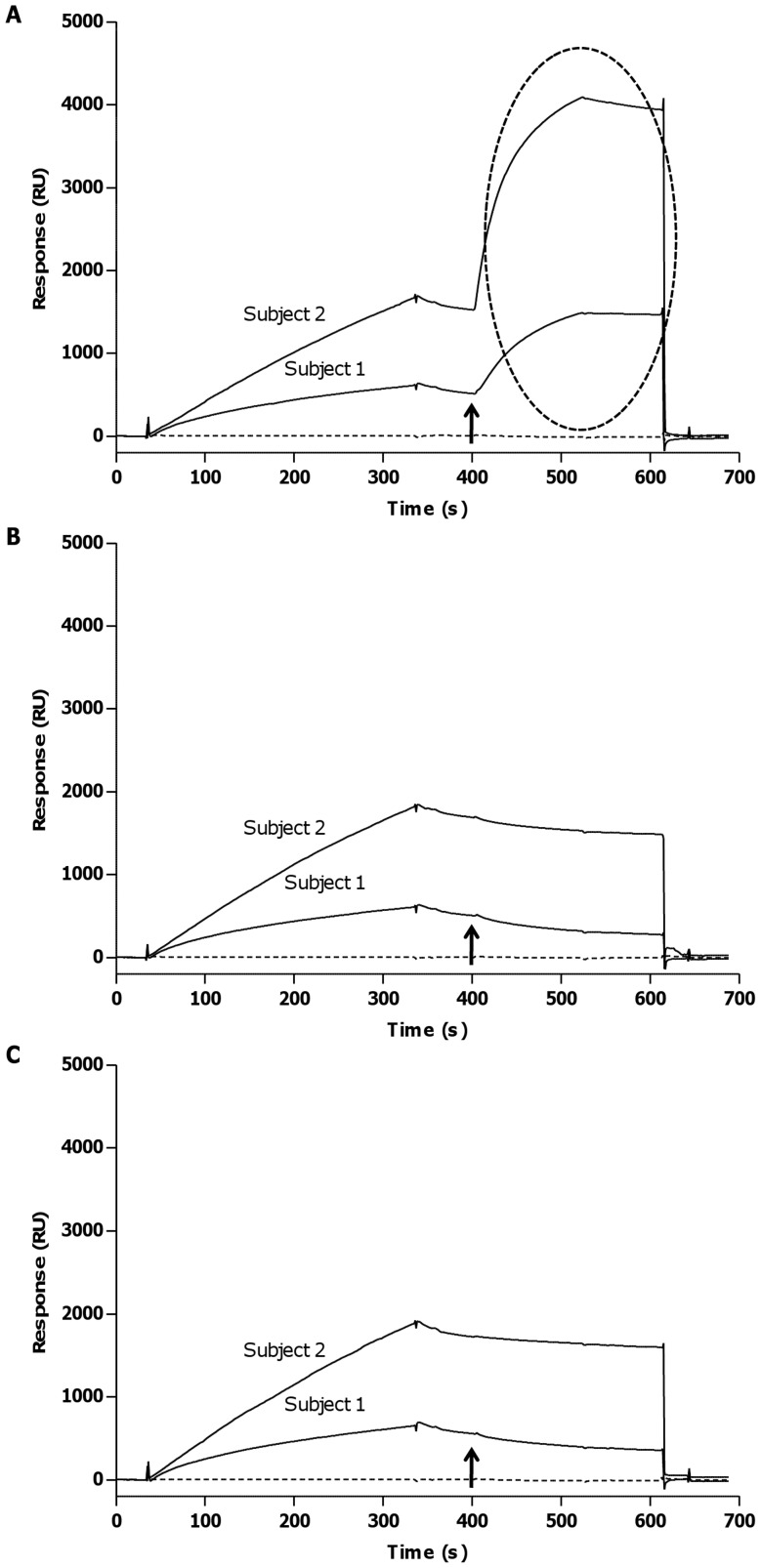
Isotyping of IA/IAA in subjects 1 and 2 by SPR technology. *A*: Sensorgrams generated by the injection of anti-human IgG. The ellipse emphasizes a positive response. *B*: Sensorgrams generated by the injection of anti-human IgM. *C*: Sensorgrams generated by the injection of anti-human IgA. The arrow indicates where in the sensorgram the anti-isotype antibodies were injected.

**Figure 4:**
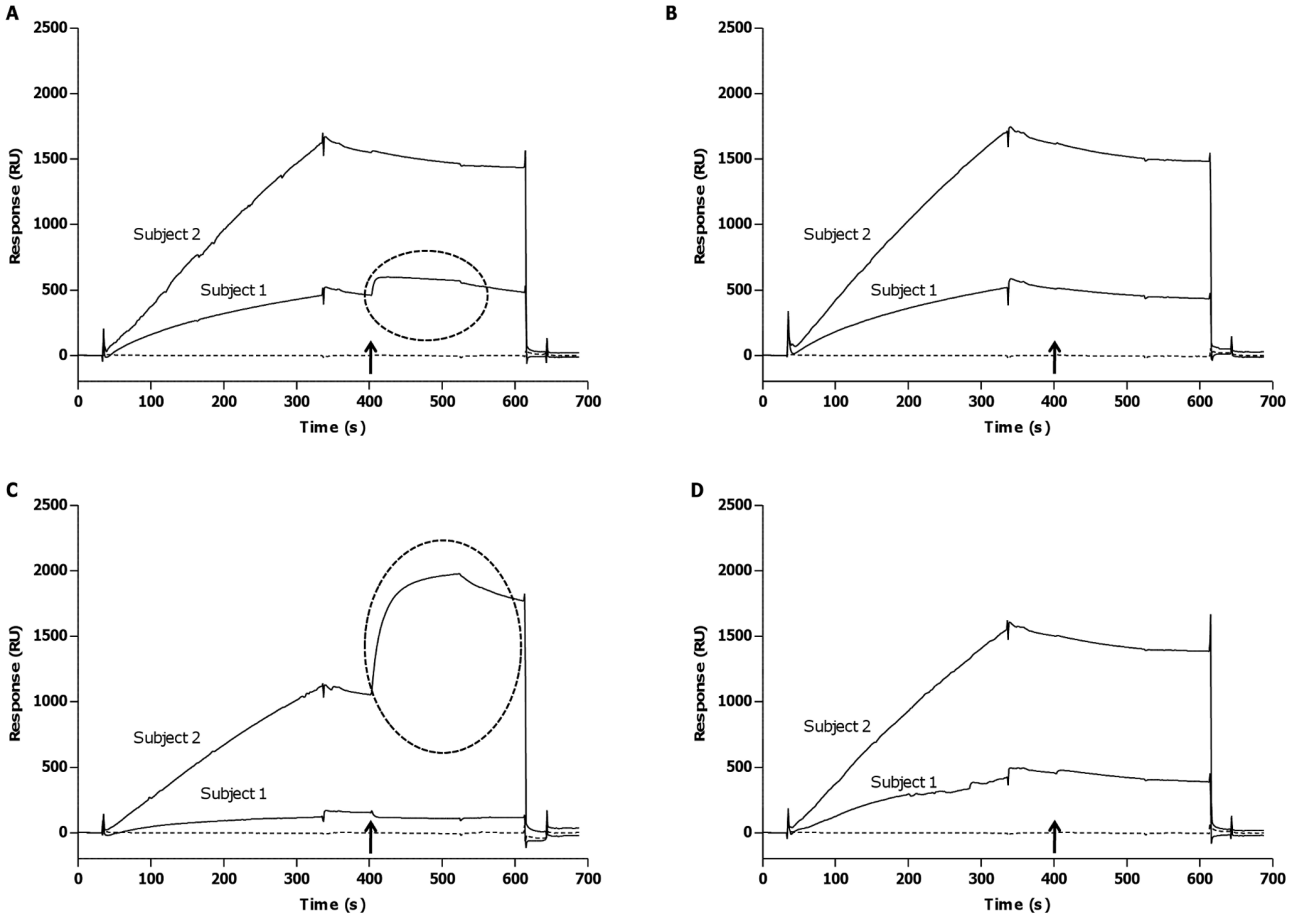
Subisotyping of IgG IA/IAA in subjects 1 and 2 by SPR technology. *A*: Sensorgrams generated by the injection of anti-human IgG_1_. *B*: Sensorgrams generated by the injection of anti-human IgG_2_. *C*: Sensorgrams generated by the injection of anti-human IgG_3_. *D*: Sensorgrams generated by the injection of anti-human IgG_4_. The ellipses in *A* and *B* emphasize a positive response. The arrow indicates where in the sensorgram the anti-subisotype antibodies were injected.

As expected for a mature secondary response, IA/IAA were IgG (961 RU and 2534 RU for subject 1 and 2, respectively), whereas the other isotypes were undetectable (Fig. 3). When analysing the IgG subisotypes involved in the humoral immune response, subject 1 had a predominant IgG_1_ response (83 RU), while subject 2 had an IgG_3_ response (875 RU) (Fig. 4).

## Discussion

It is known that a subset of insulin-treated patients with extremely high levels of insulin antibodies are insulin resistant, with mean insulin Binding Capacities greater than 216 nM (30,000 microunits of insulin/ml serum) [1]. For such patients, the species of insulin used for therapy is usually changed (e.g. human insulin to analogue insulin or vice versa and occasionally to sulphated insulin). Besides, a very rare syndrome has been described, in which patients develop extremely high levels of insulin autoantibodies after exposure to sulfhydryl containing medications (e.g. methimizole, penicillamine) [9]. This clinical entity has been termed Insulin Autoimmune Syndrome or Hirata’s syndrome [19]. These patients usually present hypoglycemia which disappears after discontinuation of the medication. In addition to this MHC-restricted syndrome (almost all with DRB1*0406), some patients have monoclonal insulin autoantibodies produced by B lymphocyte tumors.

In the present work, two particular clinical entities have been analysed whose pathologies are associated to the presence of high levels of insulin (auto)antibodies together with transient episodes of hypoglycemia. It is important to emphasize that subject 1 has type 1 diabetes with irregular control of glycemia despite receiving insulin therapy and that subject 2 is non-diabetic. Thus, it was of extreme interest to evaluate not only IA/IAA titres but also its primary interaction parameters (q and K_a_). This latter was done in order to achieve a better description of both pathologies and consequently to be able to understand the causes of the impaired glycemias.

IA/IAA titers determined by RBA, and expressed in terms of B% and SDs, were extremely high in both cases (Table1). When these values were dissected in terms of their two components (q and K_a_) by means of SPR, it was demonstrated that q values were relatively higher and K_a_ values were lower than those obtained for type 1 diabetic patients [18]. These results support the hypothesis that the IA/IAA:insulin immunocomplexes could be responsible for the uncontrolled glycemia. The alternating states of intra and interprandial hyperglycemia could be caused by a high amount of these circulating (auto)antibodies sequestering the insulin, and thus inhibiting its activity on the target tissues. In the particular case of subject 2, this level of IAA would be forcing pancreatic beta cells to produce and secrete more insulin. However, and because IAA are of low affinity and IAA:insulin immunocomplexes are of low molecular weight, there is a circulating pool of insulin bound to autoantibodies which lead to spontaneous release of high quantities of the hormone in addition to the insulin that is being released from pancreatic beta cells. All these events lead to frequent and severe symptoms of interprandial hypoglycemias.

On the other hand, subject 1 had severe signs of lipodystrophy in the insulin injection sites, problably related to the local deposit of immunocomplexes. A strong association between lipoatrophy and lipohypertrophy with insulin antibodies in children and adolescents with type 1 diabetes has been reported [20]. The authors have suggested that autoimmune phenomena with insulin play a role in the development of both abnormalities of the adipose tissue. Another study has demonstrated that patients with severe lipodystrophy are sternly insulin resistant, which can be attributed to defects in insulin action in both liver and muscle [21].

The latter signs in subject 1 had disappeared when treatment was changed to porcine insulin. Nevertheless, the alternate episodes of hyper and hypoglycemia went on along with the permanence of high levels of IA.

Despite the apparent similarity in the high levels of specific Igs, when the subisotypes of IgG involved in the humoral response were analysed, it was also demonstrated by SPR, that both patients exhibited different profiles. As expected, in subject 1,, the IA isotype was IgG_1_ accordingly to a typical type 1 diabetes [15], while in subject 2, the IAA isotype was IgG_3_ suggesting a benign monoclonal gammopathy [22], [23] associated to a pathogenic T-helper 1 response. It is well known that the isotype profile of antigen-specific autoantibodies reflects the T-helper 1/T-helper 2 (Th1/Th2) balance of the immune response, which can change during the prediabetic period. Islet cell autoimmunity may start as a non-pathogenic Th2 response to pancreatic beta-cell that gradually shifts to a pathogenic Th1 response, reflecting the maturation of the humoral immune response in individuals who progress to type 1 diabetes [24], [25]. Accordingly, the IA/IAA isotype analysis in these two patients evidenced that the autoimmune response is a complex process with different pathways that lead to the elicitation of humoral responses that lead to dissimilar physiopathological conditions.

Isotypes of insulin autoantibodies have been evaluated in the BabyDiab study and in studies from Finland [26], [27] with the observation that a broader response to insulin and strong IgG_1_ responses is associated with a somewhat greater risk of progression to diabetes. In this regard, Hoppu et al. have reported the prevalence of various isotypes of IAA in genetically susceptible young children identified from the general population. There was some heterogeneity in the IAA isotype response, but most frequently an IgG_1_-IAA response was the first to appear, whereas IgG_4_-IAA appeared later on. In addition, they have demonstrated that the initial IAA response was characterized by a dominant peak comprising IgG_1_-IAA followed by an IgG_3_-IAA response in progressors, whereas IgG_3_ dominance was not seen initially among the non-progressors [27]. These data show that genetically susceptible young children who progress to clinical type 1 diabetes are characterized by strong IgG_1_ and IgG_3_ responses to insulin, indicating a powerful insulin-specific Th1 response.

In all these reported cases, IgG subclass and isotype-specific antibodies were measured by means of a modified conventional radioimmunoassay where the protein A Sepharose precipitation was replaced by monoclonal subclass-specific antibodies linked to streptavidin agarose. It is noteworthy that only the antibodies detected with the radioassay formats are associated with type 1 diabetes [28] and that the detection with plate-binding asays is problematic [28]. Due to the latter phenomena, we have chosen SPR technology to characterize the isotypes of IA/IAA in these two clinical cases.

To sum up, it can be concluded that conventional IA/IAA assessment by RBA in these sort of patients presents serious practical limitations because it is a cuasi- quantitative assay by means of which it is only possible to determine titre as bound percentage (B%). Thus, complementary RIA/Scatchard analysis and separate isotyping tests were required to obtain a complete characterization of the humoral specific response. In this respect, it is important to highlight that with RIA/Scatchard analysis, only the concentration of specific (auto)antibodies is determined, but there is no information about the affinity.

In this sense, the present work introduces a new approach based on SPR technology for further analysis of the (auto)antibodies involved in the development of the disease. The methodology described herein, could be recommended in longitudinal studies of diabetic patients with high humoral autoimmune response and in patients where the simple positive or negative determination are not conclusive in the diagnosis of the disease.

## Supporting Information

Table S1 **Clinical and laboratory data for type 1 diabetic patients.**
(DOC)
