# Supplementary material for: Characterization of Insulin Antibodies by Surface Plasmon Resonance in Two Clinical Cases: Brittle Diabetes and Insulin Autoimmune Syndrome
Source: PLoS One. 2013 Dec 30;8(12):e84099. doi: 10.1371/journal.pone.0084099 (PMC3875527; doi:10.1371/journal.pone.0084099)
Supplement: Table S1 — Clinical and laboratory data for type 1 diabetic patients. (DOC) [file pone.0084099.s001.doc]

**Table S1.** Clinical and laboratory data for type 1 diabetic patients.

| Patients (n) | 71 |
| --- | --- |
| Mean age at diagnosis (years) | 8.3 ± 4.2 |
| Range Age (years) | 0.6 ± 19 |
| BMI | ND |
| Ketoacidosis | 38 (53%) |
| GADA + (%) | 71.8 |
| IA-2A + (%) | 66.2 |
| ZNT8A + (%) | 36.6 |
| IAA/PAA + (%) | 69.0 |
